# Regime shifts, trends, and variability of lake productivity at a global scale

**DOI:** 10.1073/pnas.2116413119

**Published:** 2022-08-22

**Authors:** Luis J. Gilarranz, Anita Narwani, Daniel Odermatt, Rosi Siber, Vasilis Dakos

**Affiliations:** ^a^Department of Aquatic Ecology, EAWAG (Swiss Federal Institute of Aquatic Science and Technology), 8600 Dübendorf, ZH, Switzerland;; ^b^Department of Surface Waters - Research and Management, EAWAG (Swiss Federal Institute of Aquatic Science and Technology), 8600 Dübendorf, ZH, Switzerland;; ^c^Department of Systems Analysis, Integrated Assessment, and Modelling, EAWAG (Swiss Federal Institute of Aquatic Science and Technology), 8600 Dübendorf, ZH, Switzerland;; ^d^Institute des Sciences de l’Evolution, Université de Montpellier, CNRS, IRD, EPHE, Montpellier, 34095, France

**Keywords:** regime shift, remote sensing, ecological stability, global change, trophic state index

## Abstract

Lakes are often described as sentinels of global change. Phenomena like lake eutrophication, algal blooms, or reorganization in community composition belong to the most studied ecosystem regime shifts. However, although regime shifts have been well documented in several lakes, a global assessment of the prevalence of regime shifts is still missing, and, more in general, of the factors altering stability in lake status, is missing. Here, we provide a first global assessment of regime shifts and stability in the productivity of 1,015 lakes worldwide using trophic state index (TSI) time series derived from satellite imagery. We find that 12.8% of the lakes studied show regime shifts whose signatures are compatible with tipping points, while the number of detected regime shifts from low to high TSI has increased over time. Although our results suggest an overall stable picture for global lake dynamics, the limited instability signatures do not mean that lakes are insensitive to global change. Modeling the interaction between lake climatic, geophysical, and socioeconomic features and their stability properties, we find that the probability of a lake experiencing a tipping point increases with human population density in its catchment, while it decreases as the gross domestic product of that population increases. Our results show how quantifying lake productivity dynamics at a global scale highlights socioeconomic inequalities in conserving natural environments.

## Significance

Lakes can change dramatically following a slow change in conditions. They can abruptly shift from being oligotrophic to eutrophic or vice versa, in what is called a regime shift. Despite the important consequences for ecosystems and human activities of abrupt shifts, we do not know how frequent they are or how they are distributed globally. To answer these questions, we analyze lake productivity dynamics of 1,015 lakes worldwide. Our results show few experienced regime shifts, yet the occurrence of observed regime shifts is increasing over time. Our analysis' global scope allows us to better understand the occurrence of regime shifts and the socioeconomic drivers associated with them. This knowledge will help manage lakes' response to global change.

Ecosystems differ greatly in their dynamics and stability over time, with some displaying relative constancy, others varying predictably over seasons, and yet others changing nonlinearly or unpredictably over longer time periods (1–3). A great deal has been written about the potential for lakes and ponds to display dramatic instabilities, including nonlinear dynamics and regime shifts in response to environmental drivers (4, 5). In particular, regime shifts triggered by anthropogenic stressors (e.g., warming or eutrophication) capture both public and scientific attention because of the severity and potential irreversibility of the incurred changes. Although regime shifts have been well documented in a number of lakes (6), the prevalence of regime shifts in lakes on a global scale remains unknown. With human water security, aquatic food security, and freshwater biodiversity at stake (7), we ignore to what extent the dynamics of lake productivity around the globe are changing. Here, we characterize regime shifts in productivity in lakes worldwide in order to quantify their prevalence. We also quantify the coefficient of variation and trend in the variability of lake productivity over time as metrics of lake stability. Finally, we test how these characteristics of lake productivity dynamics change as a result of global change drivers, such as climate change, human population density, and economic activity, to identify which drivers of lake productivity dynamics are crucial for understanding lake responses in a changing world.

Remote sensing allows the consistent and reproducible assessment of water quality in thousands of lakes worldwide (8). Remotely sensed data have been used to identify alternative trophic states in South American shallow lakes (9). In large lakes, remotely sensed phytoplankton biomass has been shown to respond to climate warming depending on the lake's trophic status (10). Although remote sensing avoids human biases when selecting study systems (11), there are limitations on the size of lakes that can be studied (*SI Appendix*, Fig. S1). Multispectral satellite images can quantify a lake's trophic state index (TSI) based on phytoplankton abundance. TSI can be used as a proxy of lake productivity, a metric typically associated with ecosystem functioning and lake dynamics (12). Whereas most work so far has focused on measuring primary production and water quality parameters for characterizing the trophic state of a lake (13–15), TSI allows a comprehensive assessment of changes in the dynamics of lake productivity that are still missing at a global scale.

Lake trophic state can respond gradually or abruptly to global change. Prolonged seasonal stratification in deep lakes due to climate warming can induce gradual long-term changes in lake productivity (16). Smooth or sharp peaks in seasonal or interannual productivity patterns are observed in lakes. The occurrence and duration of algal blooms appear to be increasing (17), and environmental change favors changes in plankton composition (18). Shallow lakes can undergo abrupt shifts to a turbid eutrophic state following the loss of aquatic macrophytes caused by an increase in nutrient loading (4). Such regime shifts can be related to a tipping point response (5). If that is the case, an increase in variance over time is a telltale sign, as it is associated with a deterioration in the stability of a lake and potentially with tipping points between alternative stable states (19).

Here, we assess lake productivity dynamics at a global scale. We investigate how TSI dynamics are changing over time, both in terms of temporal trends in the mean and variability of TSI through the study period, as well as in terms of the occurrence of regime shifts and tipping points. We use methodologically consistent water quality data of 1,015 lakes worldwide (8) to assess their trophic state based on lake TSI. From 2002 until the end of its mission in 2012, the MERIS sensor on board the Envisat satellite provided images of the Earth's surface at 300 m spatial resolution. The de-trended TSI time series allows us to compare lakes with different seasonal patterns (see *Materials and Methods*). Moreover, we obtain environmental and socioeconomic covariates to understand how climate (20), human activity in the catchments, and lake geological and geographical characteristics (21) affect global trends in lake stability, as well as the occurrence of regime shifts.

Given that aquatic ecosystems are currently affected by environmental change across the globe (20, 22–25), and that they are sensitive to extreme events (26), it is reasonable to expect that lakes are becoming less stable over time and have a higher chance of undergoing regime shifts—related or not to alternative stable states. Unfortunately, it is currently logistically impossible to conduct hundreds of whole-lake studies to estimate their ecological stability and assess whether a lake has experienced a regime shift. Instead, we can derive proxies of lake stability by measuring changes in variability (coefficient of variation and trend in variance) and by identifying abrupt changes in the pattern of fluctuations of the lake trophic state. While our approach has numerous advantages for its global scope and cost-effectiveness, the data and techniques have intrinsic limitations (*SI Appendix*). Current remote sensing sensors have resolution limits that constrain our analysis to large lakes (>6 km^2^, *SI Appendix*, Fig. S1). At the same time, pure observational data cannot conclusively determine whether a regime shift is related to shifts between alternative stable states (27, 28). Therefore, we refer to the abrupt changes observed as candidate regime shifts, and candidate tipping points, as we identify signals compatible with these processes (*Materials and Methods*). Defining true alternative states in the examined lakes would require additional evidence (28–30).

## Results

**Fig. 1.**
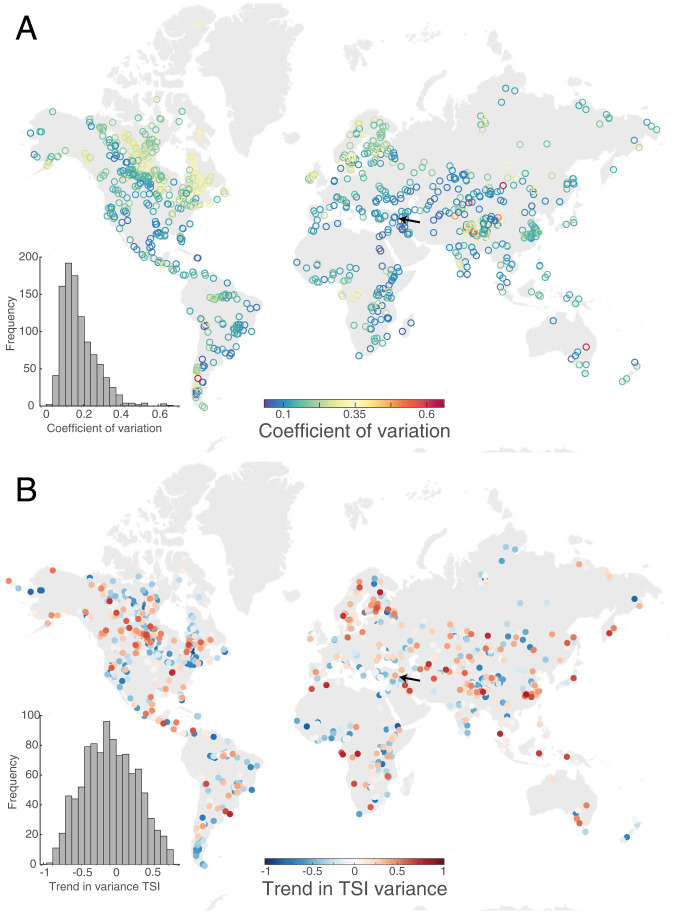
Stability of global lake productivity. Each circle represents one of the 1,015 lakes and the color represents their long-term stability, measured as the coefficient of variation in the TSI time series (*A*), and the temporal trend in stability, measured as trend in TSI variance (calculated as the Spearman rank correlation between TSI variance and time) (*B*). In each map, the insert histogram shows the distribution of measured values.

While a lake's TSI has been used to classify lakes as potentially eutrophic or oligotrophic (12), fluctuations of a lake's TSI in time could reflect how lake TSI is responding to short-term perturbations and long-term stressors (*SI Appendix*, Fig. S2 and Table S5). High temporal variability in the dynamics of an ecosystem reflects increases in the intensity and/or the number of stressors and disturbances affecting the ecosystem (2, 31). At the same time, high temporal variability is a signature of low ecological stability (32–34). Less stable ecosystems tend to show higher temporal variability measured as increased variance or coefficient of variation (35, 36). Moreover, an increase in variance over time is regarded as an indication of loss of stability due to slowly changing conditions (37) that may serve as an early warning of a nearby regime shift (19). The coefficient of variation over the entire study period measured the lake's average stability (Fig. 1*A*), while the trend in variance provided a measure of stability change (Fig. 1*B*). We found that the coefficient of variation was predominantly low, it followed a log-normal distribution (mean = 0.178, variance = 0.009), with most lakes being rather stable (Fig. 1*A*).

**Fig. 2.**
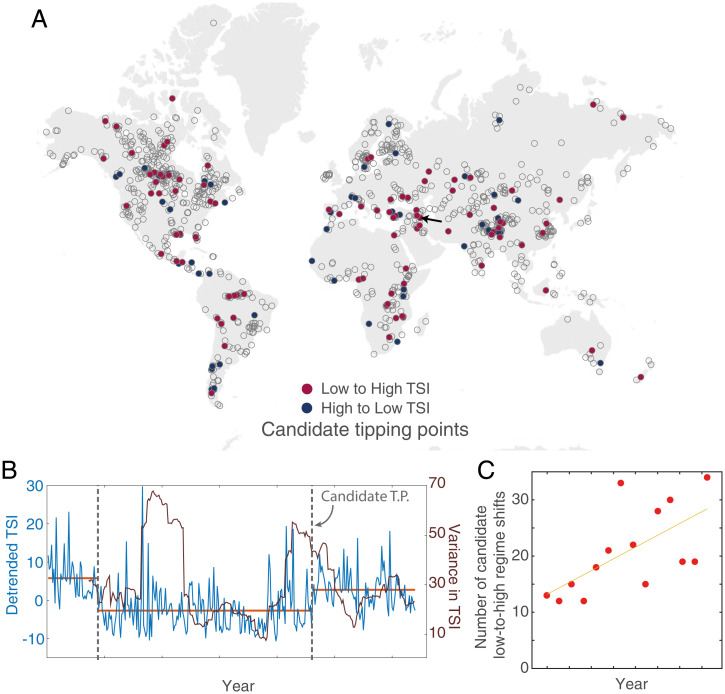
Candidate tipping points in lake productivity globally. (*A*) Each circle represents one of the 1,015 lakes. Empty circles show lakes without signs of candidate tipping points, while filled circles represent lakes that shifted from high to low TSI (blue) and lakes that shifted from low to high TSI (red) at some point in time during the study period. For each lake, we obtain the TSI time series in the period from March 2002 to April 2012, over which we calculate TSI mean (blue line) and TSI variance (red line) inside a 1-y moving window. A change-point analysis then breaks the time series when an abrupt change occurs in the mean value of TSI (see *Materials and Methods*). To be qualified as a candidate tipping point, trend in TSI variance before the change point has to be positive and significant. Moreover, we do not consider change points 2 y at the beginning and at the end of the time series. (*B*) The particular example of the change-point analysis and regime shift detection for Therthar Lake in Iraq, whose location is indicated by the arrow in (*A*). TSI values are indicated by the blue time series, while variance is indicated by the red time series. Change points in TSI mean are indicated by dotted vertical lines, but only the second one meets all the requirements to qualify as a candidate tipping point. The average TSI value between the change points is shown by horizontal orange lines. (*C*) The occurrence of candidate regime shifts is increasing over time. The yellow line shows the linear fit as a visual aide. To calculate the number of candidate regime shifts over time, the time period is discretized in bins. A sensitivity analysis shows that this trend is independent of the width of the time window over which occurrence was calculated, (*SI Appendix*, Fig. S3).

We identified candidate regime shifts in lake dynamics by first analyzing if lake TSI abruptly changed at a point in time by comparing whether the mean TSI before and after the change was significantly different (Fig. 2*B* and *Materials and Methods*). Then, to find which candidate regime shifts might result from a tipping point between alternative stable states, we tested whether the TSI variance also increased before the candidate regime shift (Fig. 2*B* and *Materials and Methods*). Such a pattern in variance trend reflects a progressive loss of resilience that has been shown to occur before regime shifts to alternative stable states (19, 38).

As a whole we found 130 lakes with candidate tipping points, which represented 12.8% of the 1,015 studied lakes (Fig. 2*A*). Lakes that shifted from low to high TSI were more numerous than lakes that shifted from high to low TSI (94 vs. 73). Also, 37 lakes shifted twice during the observation period. The relatively low reported incidence of candidate tipping points might also be linked to the stringent criteria we applied here to identify abrupt changes that could be linked to alternative states potentially caused by internal mechanisms (39). When relaxing this condition, only looking at regime shifts that would only leave the signature in the mean without influencing the variance, we find that 28% of lakes show regime shifts that could be caused by strong external stressors (e.g., storms, nutrient pollution, warming events). We found candidate regime shifts and tipping points were scattered across all kinds of biomes (Fig. 2*A*).

More interestingly, while we found candidate regime shifts only in 28% of the lakes during the study period, the occurrence of candidate regime shifts from low to high trophic states increased over time (Fig. 2*C* and Spearman ρ between 0.47 and 0.79, *SI Appendix*, Fig. S3). These results combined raise questions about the factors contributing to potential instabilities in lake phytoplankton biomass dynamics.

**Fig. 3.**
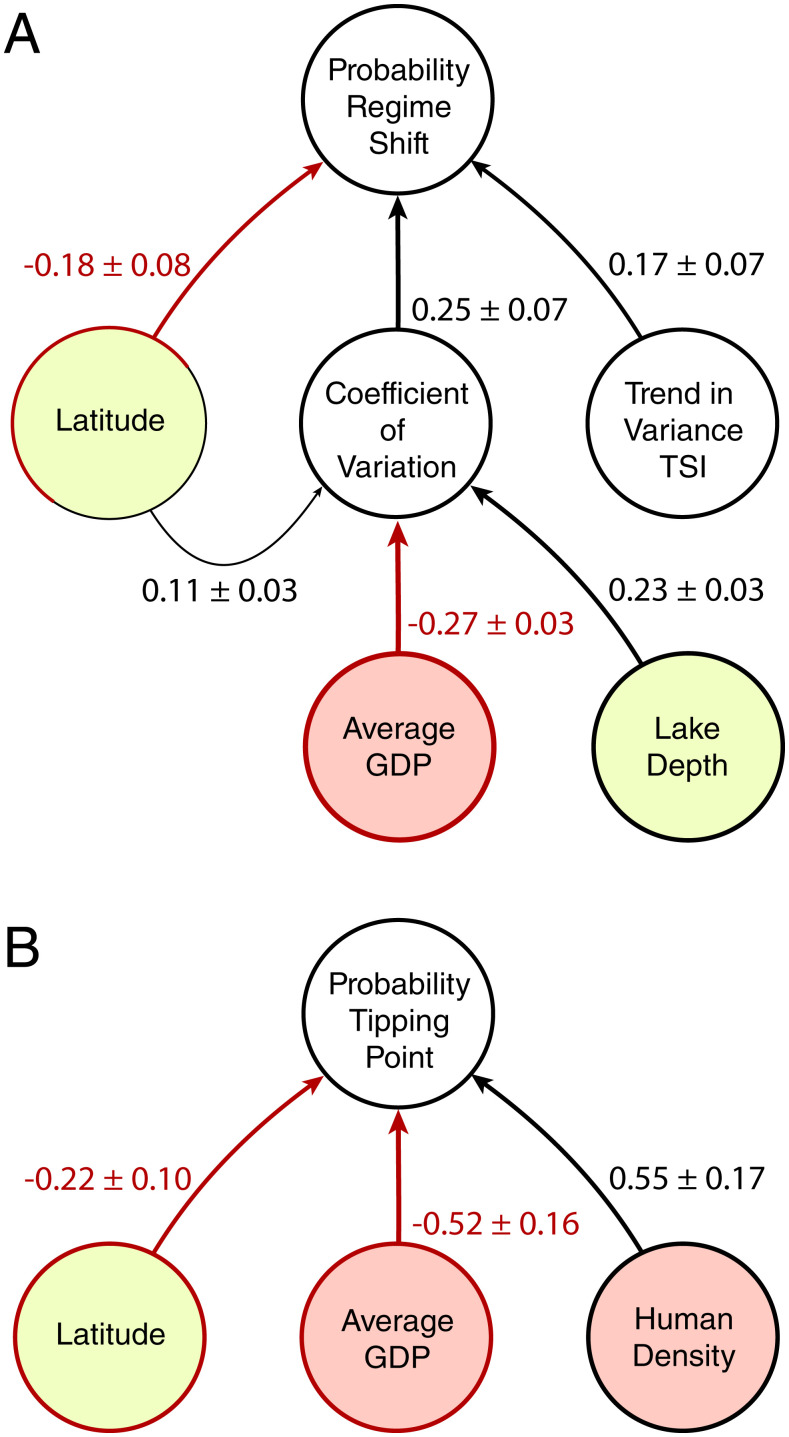
Factors affecting the probability of a lake experiencing a candidate regime shift (*A*) and a candidate tipping point (*B*). The figure represents the best supported generalized linear mixed effect models (*SI Appendix*, Tables S2–S4). Arrows between variables represent statistically significant effects of one variable on another. Line and arrow thickness and its label show the effect size, its 95% CI, and the direction of the relationships. Black lines represent positive effects, while red lines represent negative effects. Red circles represent socioeconomic variables, and yellow circles represent lakes' physical characteristics.

By controlling for a number of climatic, socioeconomic, and geophysical variables (*SI Appendix*, Table S1), we found that the probability of a lake experiencing a candidate regime shift was negatively related to its latitude, with lakes closer to the equator being less likely to experience regime shifts. We also found that candidate regime shifts were positively related to the variance trend and the coefficient of variation (Fig. 3*A* and *SI Appendix*, Table S2). At the same time, the coefficient of variation was found to be positively correlated with the lake's latitude and depth but negatively correlated with the average gross domestic product (GDP) of the human population within the catchment. In other words, wealthier populations tend to be associated with more stable lakes, which therefore have a lower probability of experiencing a regime shift. (Fig. 3*A* and *SI Appendix*, Tables S2 and S3).

The probability of a lake experiencing a candidate tipping point—when controlling for the same aforementioned variables—was also found to be negatively related to latitude. Still, the greatest explanatory power came from socioeconomic factors. While human density in the catchment increased the probability of a lake experiencing a candidate regime shift, that population's GDP decreased that probability by a similar amount (Fig. 3*B* and *SI Appendix*, Table S4).

## Discussion

Our work provides a first-ever assessment of the stability of lake productivity on a global scale. By analyzing dynamical patterns of lake TSI in remotely sensed time series from 1,015 lakes worldwide, we find seemingly limited signatures of global change negatively affecting the dynamical stability of lake productivity. This overall positive (i.e., stable) picture appears to be in contrast to the expectation that global change is having a widespread negative impact on lake ecosystems. The documented rise in lake heatwaves is expected to affect lake productivity (26), lake warming is setting off changes in the occurrence of algal blooms (25) and the stratification patterns of lakes (16). Moreover, lake productivity has been shown to abruptly respond to nutrient load changes in current and past records (40, 41). However, we find candidate tipping points in productivity only in 12.8% of lakes we study. These results seem to agree with a recent meta-analysis showing that thresholds are rare (42). Setting aside data quality issues (e.g., length of the time series, gaps, resolution, signal to noise ratio, see *SI Appendix*), that pose a priori limitations to analyzing remote-sense data, this rather low fraction might be partly explained by the fact that global change effects are not directly manifested on the dynamics of lake productivity due to time lags or due to indirect interacting effects. For instance, it has been shown that warming can increase or decrease lake phytoplankton biomass depending on whether warming is affecting productivity directly or indirectly through species interactions (10). Despite recent advances in automated monitoring of aquatic communities (43), we lack ecological data. Still, our results are in broad agreement with empirical studies that show inconsistencies in measuring stability changes in lake dynamics, especially in the vicinity of regime shifts (44, 45).

Yet, the relatively low incidence of lakes with decreasing stability signatures does not mean that lakes are insensitive to global change. By simultaneously studying climatic, geophysical, and socioeconomic lake features and their interactions, our results show that the drivers of stability are directly or indirectly linked to human-driven global change. Large lakes, like the ones studied here, tend to have larger catchments and are affected by larger human populations (the Spearman rank correlation between catchment area and lake area in the Hydrolakes database is 0.47, *P* < 0.001). Given the positive relationship, we found between a lake's probability of experiencing a candidate tipping point and population density in a catchment, as well as the wide geographical distribution of lakes experiencing negative trends in stability; we can conclude that lake instabilities might even become more common in the future as human population increases. However, the negative relationship between tipping point probability and GDP not only shows global and regional inequalities in the access to nature and the stability of the services it provides. On a positive note, this evidence suggests that as regional GDP rises, anthropogenic impacts on aquatic ecosystems may decline.

## Materials and Methods

For a full description of the methods, see *SI Appendix*, *Methods*.

### State Variables.

Lake water quality state variables are obtained from the Copernicus Global Land Service. The Copernicus Global Land Service (https://land.copernicus.eu) provides bio-geophysical products based on MERIS data, including global 10-d averages of lake TSI (46) based on a thoroughly validated chlorophyl-*a* retrieval scheme (47). TSI is obtained for 1,015 lakes between March 2002 and April 2012. We spatially averaged the pixels in each lake for each point in time to obtain a representative value of the lake's productivity. Time points were filtered out if less than 50% of the lake's surface was visible for a given 10-d period.

### Covariates.

At the lake level, we obtain information on geographic coordinates, lake depth, catchment area, lake volume, and surface water temperature. At the catchment level, we obtained information about land use cover, precipitation, human population, and subnational gross domestic product.

### Time Series Analysis: Average TSI, Trend in TSI, and TSI Coefficient of Variation.

For each lake's raw time series, we calculate average TSI, the trend in TSI measured as the Spearman rank correlation between TSI and time, and TSI coefficient of variation.

### Time Series Analysis: Trend in TSI Variance.

In order to calculate the trend in TSI variance, we first dealt with data gaps and then de-trended the data to remove the annual cycle and long-term trends.

### Dealing with Data Gaps.

Data gaps are a direct result of ice or cloud coverage. as is commonly the case for time series obtained via remote sensing.

We fill in the data gaps in the time series using a bootstrapping approach inside a 1-y-long moving window centered around the date of interest (*SI Appendix*, Fig. S5*C*). Inside the moving window, we resample the existing values of TSI. If, for example, the moving window is filled with data only 70%, and 30% is gaps, then we are doing 500 randomizations using 70% of the data (gaps or not). We, therefore, obtain 500 time series for each lake. For each data point, we obtain a mean value and its 95% CI, and we are left with no gaps but with an estimation of the uncertainty of each value.

### De-Trending.

As a next step, we de-trend the gap-filled time series by removing the annual cycle based on the whole observation time period (48). For each lake, we first select all the data from each month separately. Then, we fit a linear model to the data of each month separately. Finally, we calculate the residuals. We repeat this procedure for each month, obtaining a time series of residuals. The residuals time series becomes the time series where we calculate the trend in variance of the whole time series. In the de-trended time series, we are looking at the residuals of the TSI time series. Therefore, the trend in variance, or change points, represent deviations of the general pattern trend regardless of the seasonal pattern or gradual environmental forcing.

### Calculating Variance Trend.

High variance is commonly regarded in field data as a lack of stability (2), and it is expected to increase before a tipping point (19). In order to obtain a time series of variance (*SI Appendix*, Fig. S5*C*), variance is calculated inside a 1-y moving window as we did for filling data gaps. Calculating the variance time series over the gap-filled data ensures that all the data points in the variance time series are calculated over a population of the same size. Within that moving window, we calculate the variance over the residuals time series calculated after de-trending and bootstrapping each of the 500 aforementioned time series. Taking the average value on each time step for each of the 500 time series generates an average variance time series. The characteristic variance of each lake is then calculated as the average value of variance throughout this variance time series. This variance value is then used to calculate the coefficient of variation.

In order to calculate the trend in variance, we calculate the temporal trend of each of the 500 variance time series, as the Spearman rank correlation (ρ) between variance and time. That gives us a distribution of ρ values. In order to determine whether the trend in the mean is significant, we perform a *t* test. The *P* value of the *t* test will inform us whether the average trend in variance is significantly different from zero. The sign of the average ρ value tells us whether it is a positive or negative trend. Can you add why you did this?

### Change-Point Detection: Regime Shifts and Tipping Points.

A regime shift is defined as an abrupt change in a time series. To identify abrupt changes in the TSI time series, we first use a change-point detection algorithm (33, 34), implemented in Matlab R2019a under findchangepts, allowing for a maximum of three change points. The change-point detection is run over the de-trended gap-filled time series of TSI, as it requires an equally spaced time series free of gaps. The change-point algorithm tries to divide the time series into regions by minimizing the sum of the residual (squared) error of each region from its local mean. In the case of the TSI value, since we are interested in changes in the mean, the function to fit is an intercept representing the mean value (Fig. 1*B*). We exclude change points that occur in the first 2 or the last 2 y of the time series since we do not have enough information before or after the change point to be sure that it is a true abrupt shift.

Second, we determine whether each of the change points represents a candidate regime shift. For each change point, we calculate its abruptness. We define abruptness *a* as

$$a=\frac{\left|s\right|}{0.5(\sigma_{b}+\sigma_{a})},$$

where |s| is the absolute value of the difference between the average TSI before and after the change point, and σ_b_ and σ_a_ are the SD of TSI before and after the change point, respectively (49). We consider a candidate regime shifts those change points whose abruptness is larger than 1 (*SI Appendix*, Fig. S6). Moreover, to see if those changes represent a candidate regime shift, the mean of the state variable before and after the change point must be not only different, but they should come from different distributions. To test whether this is the case, we perform a Welch *t* test where the two populations are the time series fragments: the first fragment spans the time between the previous change point (or the beginning of the time series) and the change point of interest, and the second fragment spans the time after the change point of interest to the next change point (or the end of the time series). A candidate regime shift means that the TSI before the change point is significantly different (*P* < 0.05) from the mean after the change point. Including a statistical test to assess the differences in mean allows us to rule out those change points where there might be an abrupt change, but then the mean slowly changes back to the previous values before the change point.

Finally, we are interested in identifying regime shifts that might represent tipping points between alternative stable states. To do so, we estimate the trend in the variance of TSI only in the period before each candidate regime shift and leading up to it (as we describe in *SI Appendix, Methods*). If the trend is significantly positive, we consider a candidate regime shift as a candidate tipping point.

### Statistical Analysis.

To determine what climatic, geologic, and anthropogenic variables explain the probability of a lake experiencing a candidate regime shift or a candidate tipping point, we used generalized mixed-effects models using a binomial distribution to fit the model to the data by maximizing log-likelihood. The response variable is either 1 (the lake has experienced a candidate regime shift) or 0 (it has not). The models were structured such that the probability of experiencing a candidate regime shift or a candidate tipping point is explained by climate (temperature and precipitation), catchment and lake properties, as well as anthropogenic variables (population density and GDP). All variables used are shown in *SI Appendix*, Table S1. All fixed effects were scaled. Variables were also log-transformed when necessary. This first analysis showed that the coefficient of variation significantly increased the probability of a lake experiencing a candidate regime shift. Subsequently, a separate generalized mixed-effect model was performed to explain the coefficient of variation using the same variables input variables. The models were simplified by removing the nonsignificant variables sequentially. In all models, we used the lake's identification as a random factor, acknowledging in this way intrinsic differences between lakes.

## Supplementary Material

Supplementary File

## Data Availability

All code and data can be downloaded from https://doi.org/10.25678/0004W8. [Time series and code] data have been deposited in [ERIC] (https://doi.org/10.25678/0004W8).
