# Supplementary material for: Regime shifts, trends, and variability of lake productivity at a global scale
Source: Proc Natl Acad Sci U S A. 2022 Aug 22;119(35):e2116413119. doi: 10.1073/pnas.2116413119 (PMC9436327; doi:10.1073/pnas.2116413119)
Supplement: Supplementary File [file pnas.2116413119.sapp.pdf]

Supplementary Information for

REGIME SHIFTS, TRENDS, AND VARIABILITY OF LAKE PRODUCTIVITY AT A GLOBAL SCALE

Luis J. Gilarranz*, Anita Narwani, Daniel Odermatt, Rosi Siber, Vasilis Dakos

Corresponding author:
Email: luis.gilarranz@eawag.ch

This PDF file includes:

Supplementary methods
Figures S1 to S6
Tables S1 to S5
SI References

Supplementary Methods

S1. Data extraction

S1.1. State variables

Lake water quality state variables are obtained from the Copernicus Global Land Service (CGLS; https://land.copernicus.eu/global/). CGLS provides trophic state index and lake surface reflectance obtained by satellite remote sensing. We use the longest contiguous dataset available in CGLS, which is based on raw data acquired by the Medium Resolution Imaging Spectrometer (1) onboard the European ENVISAT satellite between March 2002 and April 2012. The database version 1.3. used in this study comprises of water quality variables for more than 1000 lakes worldwide (1). The raw data was processed with the *CaLimnos* processing chain (2–4). It facilitates data discovery, subsetting, radiometric correction, pixel identification, atmospheric correction, water quality retrieval, and aggregation. Turbidity and trophic state estimation are retrieved using an Optical Water Type classification (5). 13 OWT were predefined by clustering in situ spectral reflectance measurements (6), and the best performing algorithm was selected for each OWT and tuned for that OWT. An OWT membership score is calculated for each observed satellite pixel, and the water quality parameter retrieval for this pixel is a weighted average obtained from the algorithms that are associated with the three highest-scoring OWTs. There is a minimum size of the lakes that can be studied using these methods. For high trophic state lakes, and depending on the shape, the limit is between 3-10 Km^2. The lower the reflectance and the variable of interest, the larger the relative effect of environmental noise, which means that the size limit for oligotrophic lakes is even higher.

The CGLS products are provided as global, 10-day averaged NetCDF maps of 13 billion pixels at 300 m spatial resolution. Tabulated data was extracted from these products using the Diversity II Python library (2), which was slightly modified to match CGLS data conventions. The library uses the StatsOp of the Graph Processing Tool included in ESA's SNAP toolbox (7), and lake shapefiles that were converted from outlines provided by CGLS. In this manner, we obtain for each lake and 10-day period the spatial average, median, variance and the thresholds of the 10th and 90th percentile. If less than half of the pixels that represent the lake have been captured in a particular image (due to cloud coverage, for example), then that time step is excluded for that particular lake.

S.1.1.1. Trophic state index

The trophic state index is derived from chlorophyll retrievals from atmospherically corrected MERIS reflectance, using the conversion proposed by ref. (8). Chlorophyll is retrieved by means of four different algorithms that were identified and re-tuned for the 13 OWT, like in the case of turbidity (3). In terms of the dominant characteristics described by ref. (6), the OC2 algorithm is used for the clearest waters (https://oceancolor.gsfc.nasa.gov/atbd/chlor_a/). Waters with moderate to high levels of immersed cyanobacteria are processed with a 2-band algorithm (9), but for the most productive cyanobacteria-dominated cases, a quasi-analytical algorithm is used (10). For moderately balanced to sediment-laden, eukaryote-dominated waters, as well as for cyanobacteria scum-covered cases, another 2-band algorithm is used (11). Both 2-band algorithms use MERIS bands 7 and 9 (665 nm and 709 nm, respectively). A comprehensive matchup comparison across all OWT (n=350, R2=0.62) demonstrates a relatively high agreement, with the limitation that some of the OWT are not well represented. Note that ref. (3) also refers to Neil et al., (submitted) for validation results, but their final paper (12) recommends a slightly different set of algorithms and faulty error metrics. The dynamic switching algorithm developed in ref (12) accounts for

individual algorithms' validity ranges. They showed that chlorophyll-a retrieval uncertainties increase below 10 mg/m3, while the detection limit is below 1 mg/m3. We see no other discontinuities than the slightly lower performance at <10 mg. By binning units mg/m3 to TSI, daily to 10-day aggregates, and pixels to lake area averages, we further mitigate the decrease of signal-to-noise for lower concentrations. In other words, every 10-day lake average TSI in our time series corresponds to thousands or millions of pixels, which cancels random noise.

S1.2. Covariates at the lake level

S.1.1.1. HydroLAKES database

General knowledge about lakes, which is crucial for the investigation of ecological processes, was obtained from two different data sources. Our main data source is the HydroLAKES (13), a database of 1.4 million global lakes with a surface area of at least 10 ha, which provides a general description as well as geographic attributes such as location and area of the catchment. It also includes estimates of the lake depth, water volume, and residence time. For lakes where no HydroLAKES information was available, we used the Global Lakes and Wetlands Database (GLWD) (14). This was the case for 15 of 1016 lakes. For 4 lakes, no lake characteristics could be retrieved and were excluded from the statistical analysis.

S1.3. Covariates at the catchment level

S.1.3.1. Morphological and geographical characteristics

In order to obtain information at the catchment level, the first step is to delineate the catchments for each of the lakes. The HydroLAKES database mentioned above is a subset of HydroSHEDS, which provides not only the above-described database but also HydroBASINS, a collection of global catchments, crucial for hydrological modeling.

HydroBASINS consists of a variety of layers for the process chain for catchment delineation, from Void-filled elevation (DEM) to the final basins layer in different resolutions. It represents a seamless global coverage of hierarchically nested sub-basins.

We used HydroBASINS for the catchment delineation of the lakes in our study. Depending on the size or location of the lakes and also considering process time, different approaches are used. We used the flow direction layer in a resolution of 15 arc-seconds (approximately 500m at the equator) as input data for the lake catchment generation. If this layer was not available, we derived the catchment by aggregation of all sub-basins upstream by the hierarchical procedure. Once we obtained the catchment for each lake, we used it as a mask to obtain the catchment-level covariates at the lake level. In particular: human population, precipitation, and sub-national gross domestic product.

All spatial analysis is done in ArcGIS Desktop 10.5 (ESRI 2016) with standard GIS tools. For the catchment delineation, the extension ArcHydroTools in version 10.2 is also used with some minor adaptations in python scripts.

S.1.3.2. Human population

The world population is obtained for the year 2014 from Landscan (15), a global digital raster data in a resolution of approximately 1 km, which is the finest global population distribution data available today.

It was developed for the U.S. Department of Defense and distributed by ORNL's LandScan. We obtain the total number of populations in a catchment as well as the population density (number of people / catchment area in km^2).

S.1.3.3. Precipitation and temperature

As data sources for the precipitation, we used the Climatologies at High resolution for the Earth Land Surface Areas – CHELSA in Version 1.1 (16), a global database of the downscaled model output of temperature and precipitation estimates at a horizontal resolution of 30 arc sec (approximately 1 km). The precipitation algorithm includes orographic predictors such as wind fields, valley exposition, and boundary layer height. The resulting monthly precipitation is extracted as time series for a given catchment for each month from 2001 to 2013. The trend in precipitation and the trend in temperature for each catchment are calculated as Spearman's rank correlation between precipitation and time and temperature and time, respectively.

S.1.3.4. Sub-national gross domestic product

The Gross domestic product (GDP) is obtained from the Gridded global datasets for Gross Domestic Product (17). This is a global multiannual dataset, consistent over time and space at 5 arc-min resolution (approximately 10 km) for the 25-year period from 1990 to 2015. The GDP per capita dataset represents the average gross domestic product per capita in a given administrative area unit, whereas, for the total GDP, the above values are multiplied by a gridded population. Both datasets, available as NetCDF files, are retrieved per year and aggregated for each catchment.

S2. Statistical analysis

The variables used in the statistical analysis are reflected in SI Appendix Table S1.

S2.1. Structural equations modeling to determine variables associated with long-term trends

We used structural equations models (SEMs) to test causal hypotheses regarding the effects of climate, geographic, geologic and anthropogenic variables on the mean, variance, and the trends in both the mean and variance of TSI over time. We implemented the SEMs using the *p*SEM function in the piecewiseSEM package in R v. 4.0.2). We generated causal hypotheses by investigating the variance-covariance matrix of response variables and covariates of interest: mean temperature, mean precipitation, temporal trend in temperature, temporal trend in precipitation, lake depth, catchment area, human density, and the mean GDP of people living within the lake catchment.

We began our model selection procedures by testing causal hypotheses for variables explaining the mean TSI. We expected that the mean TSI could also be a predictor for the variance of TSI, the trend in the mean ,and the trend in the variance of TSI. To begin, we used the structured equations to test whether the mean TSI was well-predicted by climate variables (temperature and precipitation), lake properties (lake depth and catchment area), and/or anthropogenic variables (human density and average GDP). Whenever average GDP was an important predictor, we tested whether it, in turn, was well-predicted by average temperature and

human density. Similarly, whenever human density was an important predictor, we, in turn, tested whether the average temperature was an important predictor of human density. Average temperature, average precipitation, lake depth, catchment area, human density, and average GDP were all ln(+1)-transformed.

Each equation within an SEM is a linear model. We followed a progression of including missing causal links (identified by tests of directed separation, 'd-sep' tests), as well as removing non-significant pathways, repeating this procedure until all identified significant paths were included. When a path was significant, but we had no hypothesis regarding the causal relationship between the variables, we included the relationship as a correlation. We used Fischer's C statistic and the p-value to evaluate the plausibility of the models. All final models were plausible, with $p>0.05$.

We started with the following model for the mean TSI:

lm(TSIAvg ~ TemperatureAvg + PrecipitationAvg + LDepth + CatchArea + AverageGDP + HumanDensity),
lm(AverageGDP ~ TemperatureAvg + HumanDensity),
lm(HumanDensity ~ TemperatureAvg)

Model selection resulted in the following final model for the mean TSI:

lm(TSIAvg ~ TemperatureAvg + PrecipitationAvg + LDepth + CatchArea + AverageGDP),
lm(AverageGDP ~ TemperatureAvg + HumanDensity + CatchArea),
lm(HumanDensity ~ TemperatureAvg + PrecipitationAvg + CatchArea)

We followed the same procedures to select a model explaining the variance of TSI (ln(+1)-transformed), the trend in the mean TSI over time, and the trend in the variance of TSI over time. The starting models are shown below, and the final selected models are shown in Tables S1-S4.

Starting model for TSI Variance:
lm(TSIDetrendedMeanVar ~ TSIAvg + TemperatureAvg + PrecipitationAvg + LDepth + CatchArea + AverageGDP + HumanDensity,
lm(TSIAvg ~ TemperatureAvg + PrecipitationAvg + LDepth + CatchArea + AverageGDP,
lm(AverageGDP ~ TemperatureAvg + CatchArea + HumanDensity,
lm(HumanDensity ~ TemperatureAvg + PrecipitationAvg + CatchArea

We used the trend in TSI and trends in temperature and precipitation as predictors for the trend mean and trend in the variance of TSI.

Starting model for trend in mean TSI:

lm(TSIMeanTrend ~ TSIAvg + TemperatureAvg + PrecipitationAvg + TemperatureTrend + PrecipitationTrend + LDepth + CatchArea + AverageGDP + HumanDensity,
lm(TSIAvg ~ TemperatureAvg + PrecipitationAvg + LDepth + CatchArea + AverageGDP,
lm(AverageGDP ~ TemperatureAvg + CatchArea + HumanDensity,
lm(HumanDensity ~ TemperatureAvg + PrecipitationAvg + CatchArea

Starting model for trend in variance TSI:

lm(TSIDetrendedVarTrend ~ TSIAvg + TSIDetrendedMeanVar + TSIMeanTrend + TemperatureAvg + PrecipitationAvg + TemperatureTrend + PrecipitationTrend + LDepth + CatchArea + AverageGDP + HumanDensity,
lm(TSIMeanTrend ~ TSIAvg + TemperatureAvg + PrecipitationAvg,
lm(TSIDetrendedMeanVar ~ TSIAvg + LDepth + CatchArea + AverageGDP,
lm(TSIAvg ~ TemperatureAvg + PrecipitationAvg + LDepth + CatchArea + AverageGDP,
lm(AverageGDP ~ TemperatureAvg + PrecipitationAvg + CatchArea + HumanDensity,
lm(HumanDensity ~ TemperatureAvg + PrecipitationAvg + CatchArea

The results are shown in Table S5.

S3. Limitations

S3.1. Spatial resolution

There are several limitations to be considered and improved upon in the future. The first one is the spatial resolution of the remote sensing data. This translates into a bias towards large lakes (Fig. S1*B*) when compared to other reference lake datasets like Hydrolakes. Since lake area and depth are correlated, the spatial resolution limit also translates into a bias towards deep lakes (Fig. S1*C*), although this bias is not as strong as in the case of lake area.

S3.2. Length of the time series

Even though we are using the longest time series available, they might be too short to show signals of instability in some lakes. For this reason, we cannot describe a lake as stable, only to report the abrupt changes and signs of instability that we observe within the period covered in these time series.

S3.3. Data gaps

For the change-point detection algorithm, a continuous time series sampled at regular intervals is a pre-requisite. Meris sensors work on the optical range and cannot penetrate clouds. Therefore, the number of gaps in the time series depends on how much cloud coverage or ice a lake experiences. Lakes in high latitudes are more prone to have long periods without data, particularly during winter. The Spearman's rank

correlation between the number of missing data points and the absolute value of latitude is 0.43, with a p-value < 0.001.

During the gap-filling routine, when the data within a moving window was scarce, the uncertainty around the gap-filling was such that it is increasingly difficult to find significant trends in the data. The correlation between the number of data gaps and the significance of the slope between two change points is -0.47, p-value < 0.001).

S3.4. Finding signatures compatible with fold bifurcations in observational data

Pure observational data cannot conclusively determine whether a regime shift is the result of a fold bifurcation. Hysteresis could not be detected without knowing what the control parameter might be and quantifying it in situ, the control parameter might not continue to change after the shift, or the system might not relax faster than the sampling frequency. Therefore, we refer to the abrupt changes observed as candidate regime shifts and candidate tipping points.

S3.5. Chosen state variable

Lakes might experience a regime shift in another variable other than TSI within the study period, like turbidity or the population of specific species.

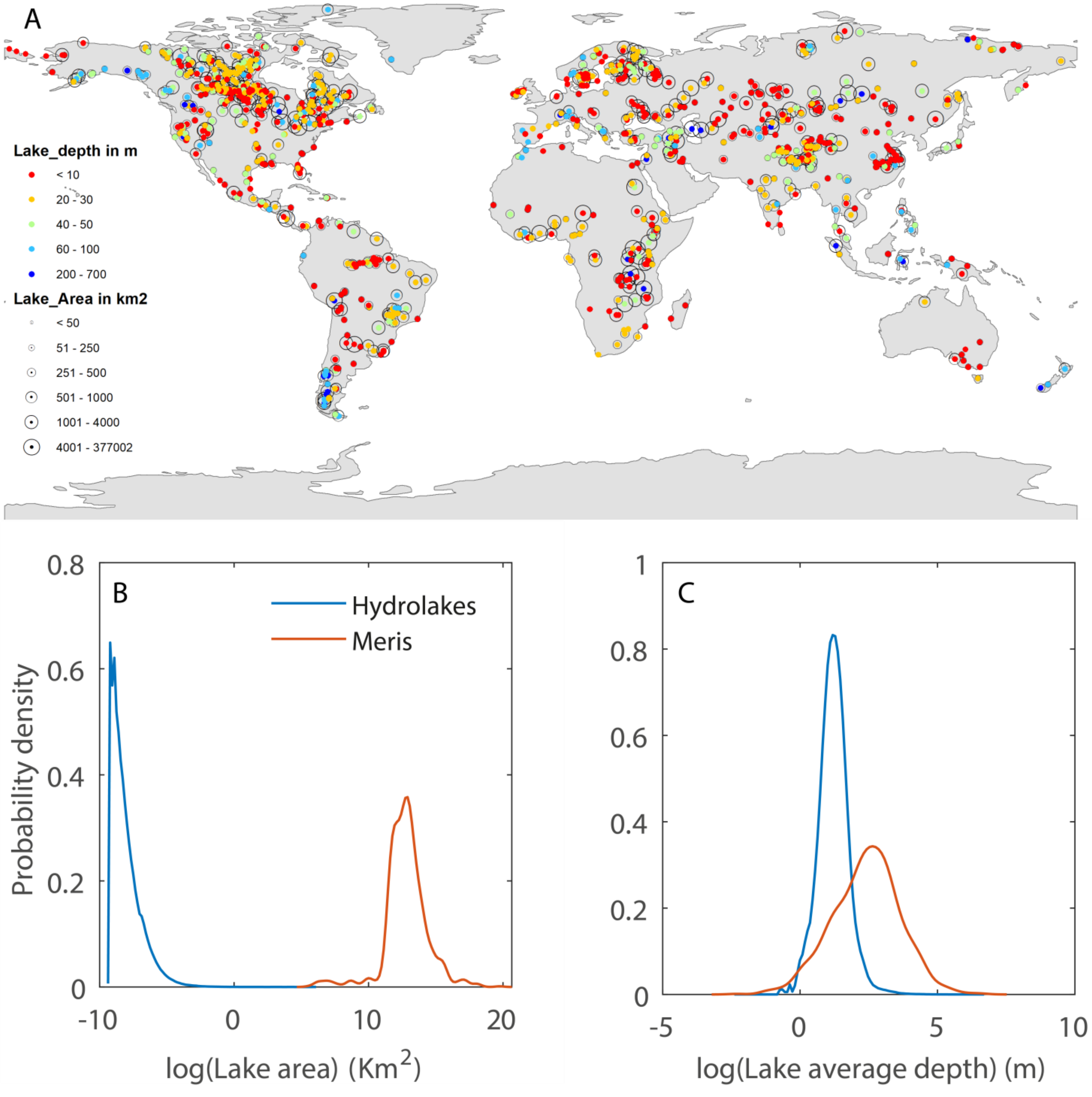

Fig. S1. *A*: Depth and area of each of the 1015 lakes. *B* and *C* show biases in lake size and depth of the lakes in this study (Meris) compared to a global database of lakes (Hydrolakes) with 1.4 million lakes. The spatial resolution of the Meris sensor constrains data acquisition to lakes larger than 6Km2. This also results in a slight bias in average lake depth.

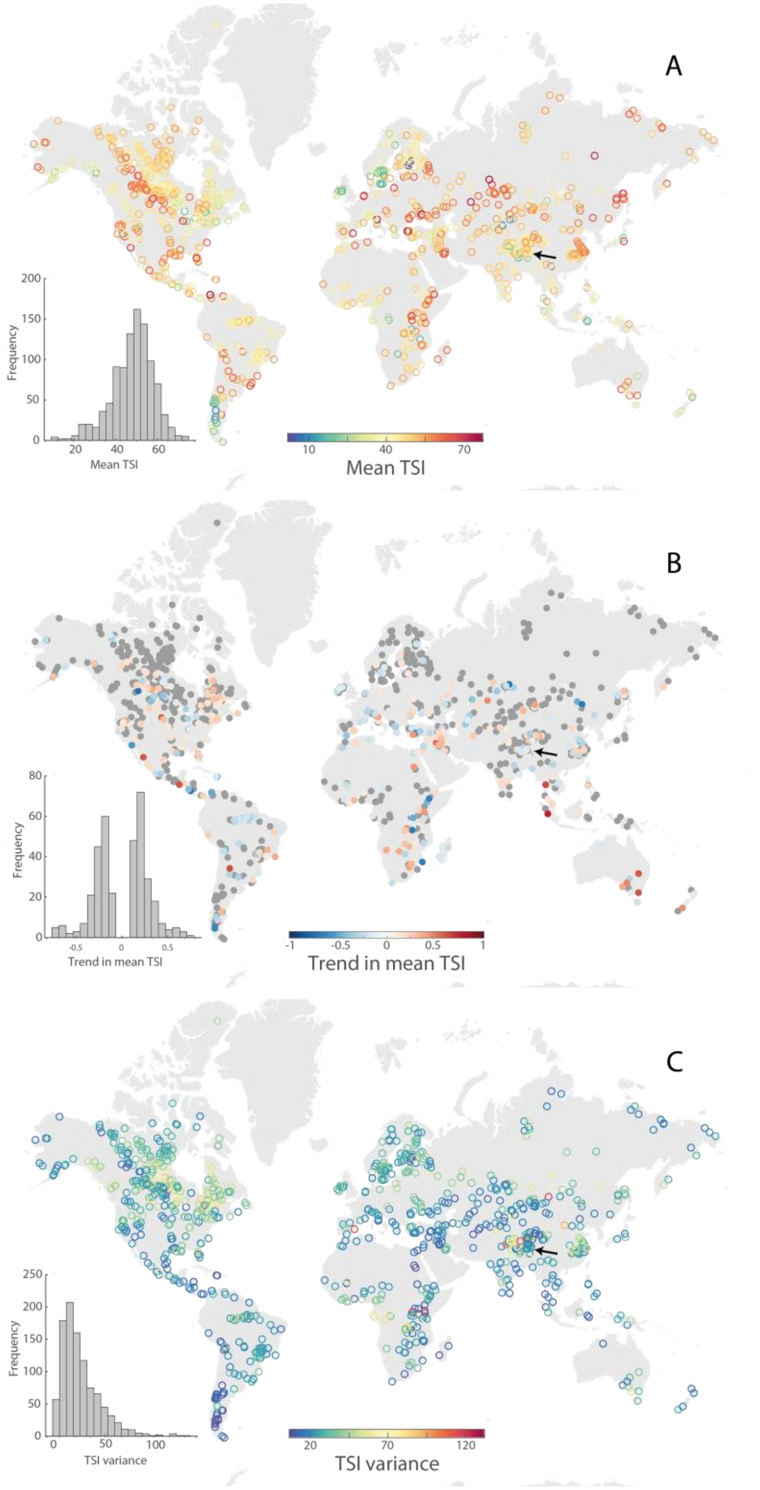

Fig. S2. Global lake productivity and its temporal trend. Each dot represents one of the 1015 lakes, and the color represents their mean productivity, measured as trophic state index (*A*), and the trend in productivity, measured as the trend in mean TSI, quantified as the Spearman's rank correlation between mean TSI and time (*B*). Non-significant trends are shown in grey. Positive trends are shown in different shades of red, while negative trends are blue. 18.4% of the lakes show positive trends in TSI, while 16.9% of lakes show negative trends. The fraction of lakes with non-significant trends was 64.7%. In both maps, the insert shows the histogram of significant trends. (*C*) Stability of global lake productivity measured as variance in the de-trended TSI time series.

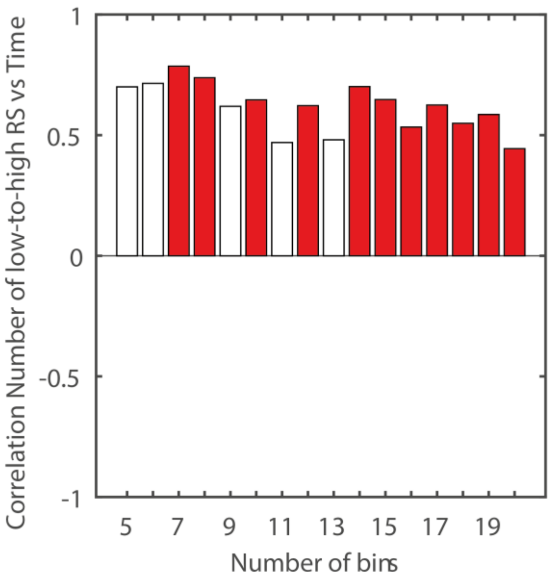

Fig. S3. Sensitivity analysis. This figure shows that the frequency of candidate regime shifts from low to high TSI is increasing over time regardless of the number of bins in which we partition the time series. Although not for every partition, the correlation is significant (Spearman's rank correlation p-val < 0.05, orange bars), the pattern is consistent. All correlation coefficients are positive and similar in magnitude. Moreover, this can also be understood as that the time between two candidate regime shifts is decreasing over time. Such correlation (time between candidate regime shifts in the dataset vs. time) would not require a sensitivity analysis. We find that correlation to be significant (p-val < 0.01) Spearman's rank correlation of -0.17, confirming that, over the length of the time series, candidate regime shifts from low to high TSI have become more frequent over time. The temporal trends in the total number of candidate regime shifts (independent of shift direction), and candidate regime shifts from high to low TSI are shown in Fig. S4.

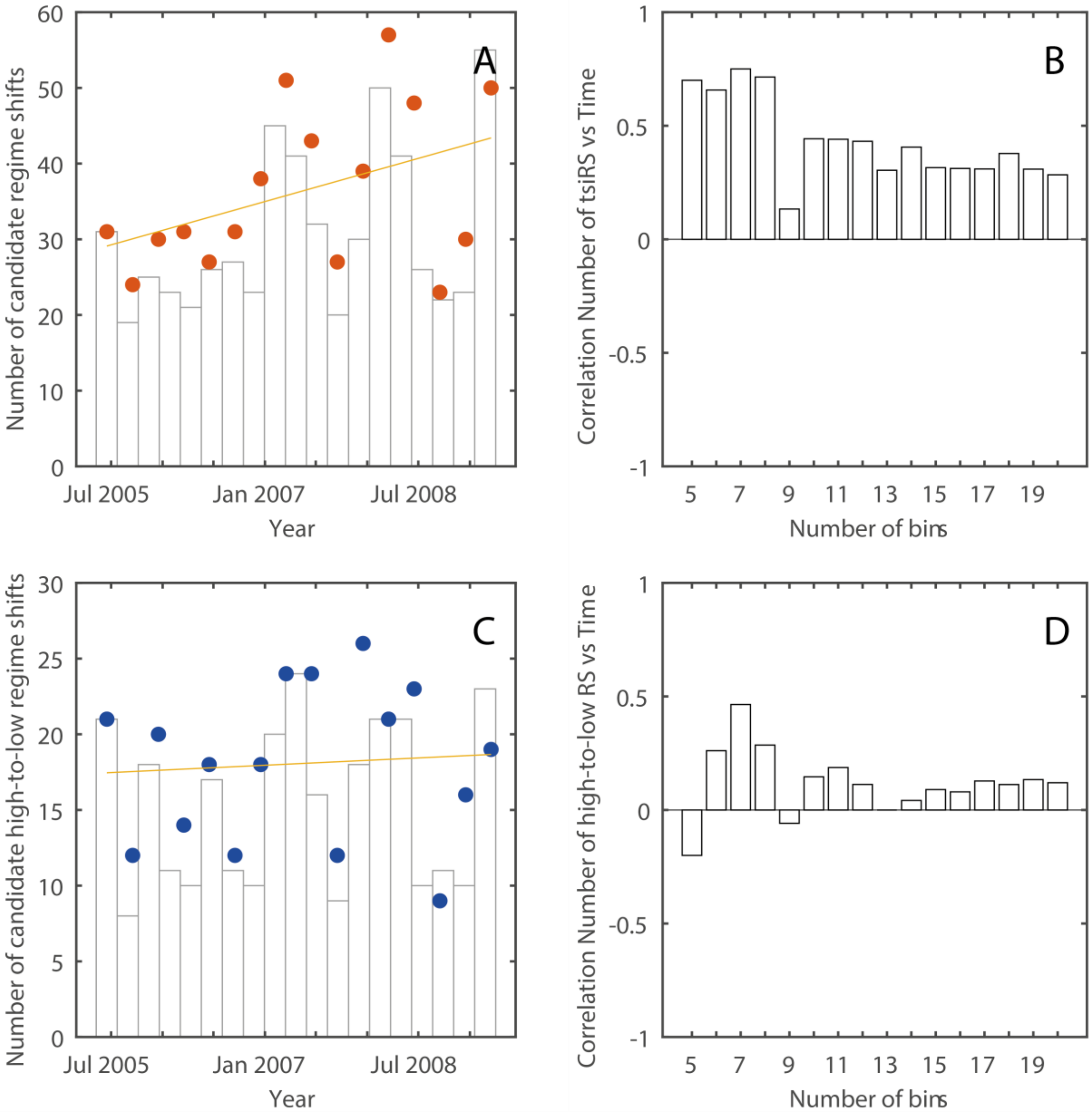

Fig. S4. Panels *A* and *C* are equivalent to figure 2*C* in the main text but for all candidate regime shifts (*A*) and for lakes that go from high to low TSI as a result of the shift (*C*). Panels *B* and *D* are the sensitivity analysis equivalent to Figure S3. It is clear that the positive (although not significant) trend in the total number of candidate regime shifts is driven by the increase in candidate regime shifts from low to high TSI shown in figure S3. No temporal trends were observed in the number of candidate tipping points.

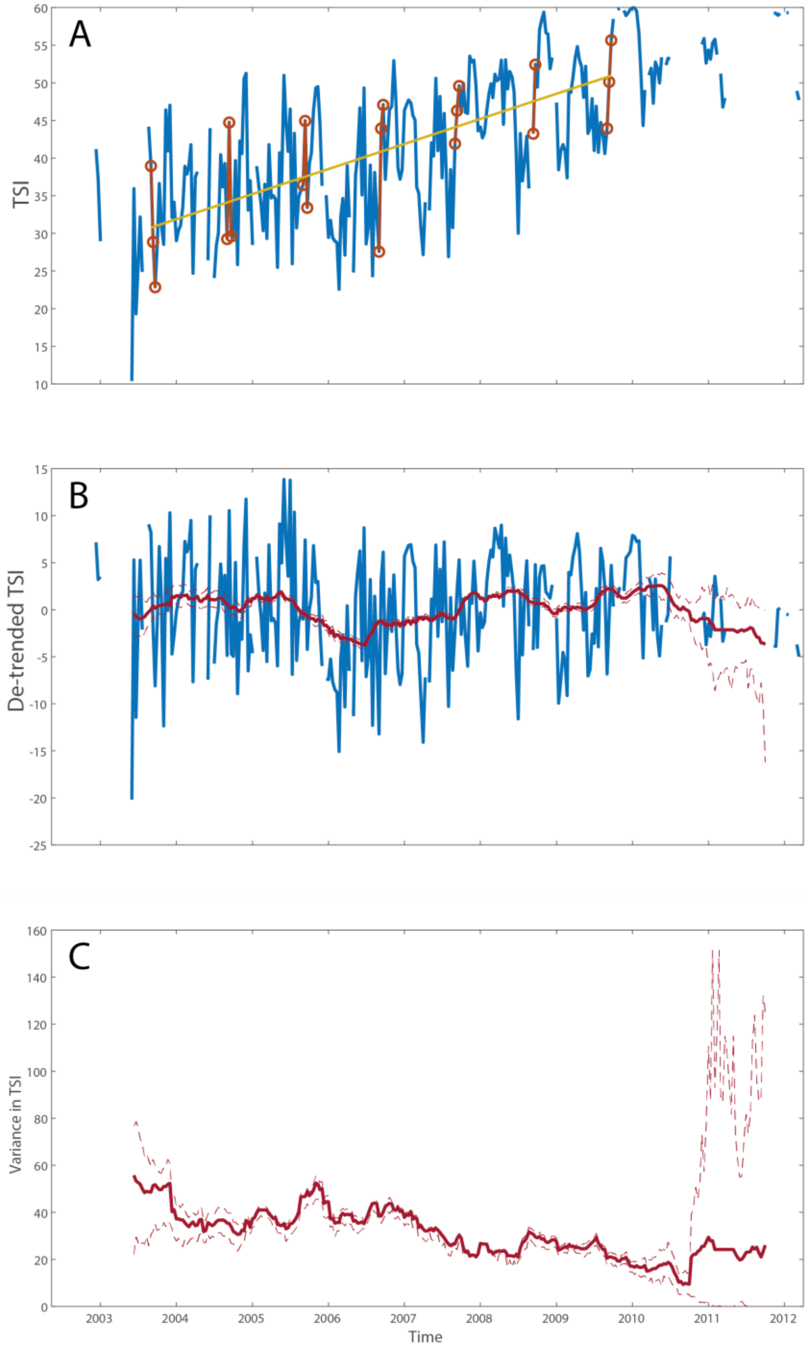

Fig. S5. Detrending and moving average. (*A*) shows the TSI time series of a lake, where the data in red represents the data of a certain month (September), and the line shows the linear fit of the data from that particular month. (*B*) shows the de-trended time series. The solid red line shows the mean de-trended TSI inside a 1-year moving window. (*D*) Shows the Variance in TSI inside a 1-year moving window (*solid red line*). The dotted lines show the 95% confidence intervals. The lake shown in this example is lake Toshka.

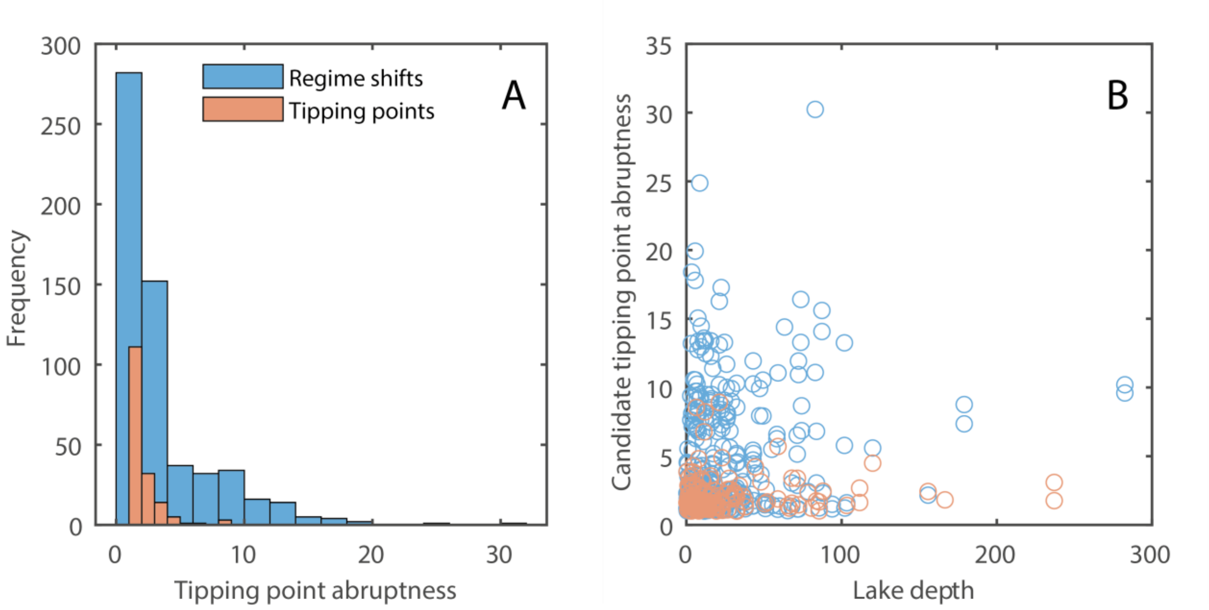

Fig. S6. Candidate regime shifts and tipping point abruptness. (*A*) Histogram of abruptness values for the change points classified as candidate regime shifts (blue) and candidate tipping points (orange). (*B*) Relationship between lake depth and abruptness. Blue circles represent candidate regime shifts, while orange circles represent candidate tipping points.

Table S1. List of variables and covariates.

Abbreviation	Meaning
TSIAvg	Average TSI before de-trending
TSIMeanTrend	Trend in mean TSI before de-trending
TSIDetrendedMeanVar	Mean variance of the de-trended TSI time series
TSIDetrendedVarTrend	Trend in the variance above
CVraw	Coefficient of Variation
Latitude	Latitude
Longitude	Longitude
LDepth	Lake's maximum depth
CatchArea	Catchment area
LVolume	Lake's volume
AverageGDP	Average GDP of the human population inside the lake's catchment
AverageGDPperCap	Average GDP per capita
HumanDensity	Human density in the catchment

Table S2: Best model to explain the probability of a lake experiencing a candidate regime shift in TSI (RSTSI is a binary vector indicating whether a lake has experienced a candidate regime shift, 1, or not, 0). After model selection, the final binomial model takes the following form: *RSTSI ~ 1 + TSIDetrendedVarTrend + CVraw + Latitude+ (1 | LakeID)*

	Effect	Std. Error	t-value	DF	p-value
(Intercept)	-0.683	0.074	-9.23	835	<0.001
TSIDetrendedVarTrend	0.174	0.074	2.34	835	0.019
CVraw	0.246	0.073	3.34	835	<0.001
Latitude	-0.177	0.074	-2.39	835	0.017

Table S3: Best model to explain the coefficient of variation in a lake. After model selection, the final model takes the following form: *CVraw ~ 1 + Latitude + log(LDepth) + log(AverageGDP) + (1 | LakeID)*

	Effect	Std. Error	t-value	DF	p-value
(Intercept)	0	0.031	0	835	1
Latitude	0.108	0.032	3.36	835	<0.001
log(LDepth)	0.230	0.032	7.20	835	<0.001
log(Average GDP)	-0.266	0.032	-8.26	835	<0.001

Table S4: Best model to explain the probability of a lake experiencing a candidate tipping point in TSI (TPTSI is a binary vector indicating whether a lake has experienced a candidate tipping point, 1, or not, 0). After model selection, the final binomial model takes the following form: *TPTSI ~ 1 + Latitude + log(AverageGDP) + log(HumanDensity) + (1 | LakeID)*

	Effect	Std. Error	t-value	DF	p-value
(Intercept)	-1.771	0.102	-17.43	835	<0.001
Latitude	-0.220	0.097	-2.26	835	0.023
log(Average GDP)	-0.524	0.162	-3.24	835	0.001
log(Human density)	0.547	0.168	3.26	835	0.001

Table S5: Interactions between climatic, socioeconomic, and geophysical drivers and lake productivity characteristics. The table represents the best-supported structural equations models describing causal hypotheses explaining lake stability (TSI variance), and the mean lake productivity (mean TSI). No significant predictors of the trend in lake stability (TSI Trend in Variance) were found. We observed significant relationships between lakes' mean TSI and TSI variance, which implies that more productive lakes are more stable. At the same time, lake mean productivity was positively correlated with human density, but negatively affected by precipitation. Lake depth also had a strong negative effect on mean productivity, consistent with theoretical expectations (18).

The test of direct separation gave a Fisher's C of 13.555 with a P-value of 0.483 on 14 degrees of freedom. The final model takes the following form:

TSIDetrendedMeanVar ~ TSIAvg + LDepth + HumanDensity + TemperatureTrend + PrecipitationTrend
TSIAvg ~ PrecipitationAvg + LDepth + HumanDensity
HumanDensity ~ TemperatureAvg + PrecipitationAvg + CatchArea
HumanDensity ~~ TemperatureTrend
TSIAvg ~~ TemperatureTrend
TSIAvg ~~ PrecipitationTrend

Where ~~ indicates correlations between two variables where there is no hypothesis about the causality of those paths.

Response	Predictor	Estimate	Std.Error	DF	Crit.Value	P.Value	Std.Estimate	
TSIDetrendedMeanVar	TSIAvg	-0.0108	0.0026	947	-4.1597	0	-0.1529	***
TSIDetrendedMeanVar	LDepth	-0.0574	0.0234	947	-2.4577	0.0142	-0.09	*
TSIDetrendedMeanVar	HumanDensity	-0.0496	0.011	947	-4.528	0	-0.1456	***
TSIDetrendedMeanVar	TemperatureTrend	-0.6598	0.1896	947	-3.4806	0.0005	-0.1102	***
TSIDetrendedMeanVar	PrecipitationTrend	-0.8052	0.2057	947	-3.9144	0.0001	-0.1256	***
TSIAvg	PrecipitationAvg	-1.3486	0.2635	949	-5.1182	0	-0.1501	***
TSIAvg	LDepth	-4.6409	0.2509	949	-18.4955	0	-0.516	***
TSIAvg	HumanDensity	0.5404	0.1402	949	3.8554	0.0001	0.1124	***
HumanDensity	TemperatureAvg	42.1169	2.5751	949	16.3557	0	0.4865	***
HumanDensity	PrecipitationAvg	0.2505	0.0546	949	4.5862	0	0.1341	***
HumanDensity	CatchArea	0.0822	0.0283	949	2.9041	0.0038	0.0795	**
~~HumanDensity	~~TemperatureTrend	-0.1166	-	953	-3.6177	0.0002	-0.1166	***
~~TSIAvg	~~TemperatureTrend	0.0819	-	953	2.5319	0.0058	0.0819	**
~~TSIAvg	~~PrecipitationTrend	-0.1011	-	953	-3.1334	0.0009	-0.1011	***

Individual R-squared

Response	Marginal R^2
TSIDetrendedMeanVar	0.07
TSIAvg	0.28
HumanDensity	0.33

SI References

1. M. Rast, J. L. Bezy, S. Bruzzi, The ESA Medium Resolution Imaging Spectrometer MERIS a review of the instrument and its mission. *Int. J. Remote Sens.* **20**, 1681–1702 (1999).
2. D. Odermatt, O. Danne, P. Philipson, C. Brockmann, Diversity II water quality parameters from ENVISAT (2002–2012): a new global information source for lakes. *Earth Syst. Sci. Data* **10**, 1527–1549 (2018).
3. S. Simis, K. Stelzer, D. Müller, “Algorithm Theoretical Basis Document Lake Water Quality 300m and 1km Products” (2018).
4. K. Stelzer, S. Simis, D. Müller, “Product User Manual Lake Water Quality 300m and 1km Products” (2018).
5. T. S. Moore, M. D. Dowell, S. Bradt, A. Ruiz Verdu, An optical water type framework for selecting and blending retrievals from bio-optical algorithms in lakes and coastal waters. *Remote Sens. Environ.* **143**, 97–111 (2014).
6. E. Spyrakos, *et al.*, Optical types of inland and coastal waters. *Limnol. Oceanogr.* **63**, 846–870 (2018).
7. M. Zühlke, *et al.*, SNAP (Sentinel Application Platform) and the ESA Sentinel 3 Toolbox in *Proc. Sentinel-3 for Science Workshop*, ESA Special Publication., L. Ouwehand, Ed. (ESA, 2015), p. 4.
8. R. E. Carlson, A trophic state index for lakes. *Limnol. Oceanogr.* **22**, 361–369 (1977).
9. A. A. Gilerson, *et al.*, Algorithms for remote estimation of chlorophyll-a in coastal and inland waters using red and near infrared bands. *Opt. Express* **18**, 24109–24125 (2010).
10. S. Mishra, D. R. Mishra, Z. Lee, Bio-Optical Inversion in Highly Turbid and Cyanobacteria-Dominated Waters. *IEEE Trans. Geosci. Remote Sens.* **52**, 375–388 (2014).
11. H. J. Gons, Effect of a waveband shift on chlorophyll retrieval from MERIS imagery of inland and coastal waters. *J. Plankton Res.* **27**, 125–127 (2004).
12. C. Neil, E. Spyrakos, P. D. Hunter, A. N. Tyler, A global approach for chlorophyll-a retrieval across optically complex inland waters based on optical water types. *Remote Sens. Environ.* **229**, 159–178 (2019).
13. M. L. Messager, B. Lehner, G. Grill, I. Nedeva, O. Schmitt, Estimating the volume and age of water stored in global lakes using a geo-statistical approach. *Nat. Commun.* **7**, 13603 (2016).
14. B. Lehner, P. Döll, Development and validation of a global database of lakes, reservoirs and wetlands. *J. Hydrol.* **296**, 1–22 (2004).
15. E. A. Bright, A. N. Rose, M. L. Urban, LandScan 2015 (2016).
16. D. N. Karger, *et al.*, Climatologies at high resolution for the earth's land surface areas. *Sci. Data* **4**, 170122 (2017).
17. M. Kummu, M. Taka, J. H. A. Guillaume, Gridded global datasets for Gross Domestic Product and Human Development Index over 1990–2015. *Sci. Data* **5**, 180004 (2018).
18. S. R. Carpenter, Lake geometry: Implications for production and sediment accretion rates. *J. Theor. Biol.* **105**, 273–286 (1983).
